# Virological Failure and HIV-1 Drug Resistance Mutations among Naive and Antiretroviral Pre-Treated Patients Entering the ESTHER Program of Calmette Hospital in Cambodia

**DOI:** 10.1371/journal.pone.0105736

**Published:** 2014-08-28

**Authors:** Hubert Barennes, Stéphanie Guillet, Setha Limsreng, Sovanvatey Him, Janin Nouhin, Chanroeurn Hak, Chanvatey Srun, Gerald Viretto, Vara Ouk, Jean Francois Delfraissy, Olivier Ségéral

**Affiliations:** 1 Agence Nationale de Recherche sur le VIH et les Hepatites (ANRS), Phnom Penh, Cambodia; 2 ISPED, Centre INSERM U897-Epidemiologie-Biostatistique, Univ. Bordeaux, Bordeaux, France; 3 Epidemiology Unit, Pasteur Institute, Phnom Penh, Cambodia; 4 Assistance Publique - Hôpitaux de Paris (AP-HP), Département de Médecine Interne et Immunologie Clinique, Centre Hospitalier Universitaire de Bicêtre, Le Kremlin-Bicêtre, France; 5 Hospital Calmette, Phnom Penh, Cambodia; 6 Ensemble pour une Solidarité Thérapeutique Hospitalière En Réseau (ESTHER), Phnom Penh, Cambodia; 7 HIV/Hepatitis Unit, Pasteur Institute, Phnom Penh, Cambodia; 8 Université Paris Diderot, Sorbonne Paris Cité, Paris, France; University of Athens, Medical School, Greece

## Abstract

**Introduction:**

In resource limited settings, patients entering an antiretroviral therapy (ART) program comprise ART naive and ART pre-treated patients who may show differential virological outcomes.

**Methods:**

This retrospective study, conducted in 2010–2012 in the HIV clinic of Calmette Hospital located in Phnom Penh (Cambodia) assessed virological failure (VF) rates and patterns of drug resistance of naive and pre-treated patients. Naive and ART pre-treated patients were included when a Viral Load (VL) was performed during the first year of ART for naive subjects or at the first consultation for pre-treated individuals. Patients showing Virological failure (VF) (>1,000 copies/ml) underwent HIV DR genotyping testing. Interpretation of drug resistance mutations was done according to 2013 version 23 ANRS algorithms.

**Results:**

On a total of 209 patients, 164 (78.4%) were naive and 45 (21.5%) were ART pre-treated. Their median initial CD4 counts were 74 cells/mm^3^ (IQR: 30–194) and 279 cells/mm^3^ (IQR: 103–455) (p<0.001), respectively. Twenty seven patients (12.9%) exhibited VF (95% CI: 8.6–18.2%), including 10 naive (10/164, 6.0%) and 17 pre-treated (17/45, 37.8%) patients (p<0.001). Among these viremic patients, twenty-two (81.4%) were sequenced in reverse transcriptase and protease coding regions. Overall, 19 (86.3%) harbored ≥1 drug resistance mutations (DRMs) whereas 3 (all belonging to pre-treated patients) harbored wild-types viruses. The most frequent DRMs were M184V (86.3%), K103N (45.5%) and thymidine analog mutations (TAMs) (40.9%). Two (13.3%) pre-treated patients harbored viruses that showed a multi-nucleos(t)ide resistance including Q151M, K65R, E33A/D, E44A/D mutations.

**Conclusion:**

In Cambodia, VF rates were low for naive patients but the emergence of DRMs to NNRTI and 3TC occurred relatively quickly in this subgroup. In pre-treated patients, VF rates were much higher and TAMs were relatively common. HIV genotypic assays before ART initiation and for ART pre-treated patients infection should be considered as well.

## Introduction

Anti retroviral therapy (ART) availability has considerably increased in resource-limited settings. However, the emergence and spread of high levels of HIV-1 drug resistance could compromise the effectiveness of national HIV treatment programmes [1]. In resource limited settings, the majority of patients are switched to a second-line ART regimen according to WHO clinical and immunologic criteria, due to lack or paucity of viral load (VL) monitoring [2], [3]. These criteria lack both sensitivity and specificity and are associated with unacceptable treatment failure misclassification [4], [5]. Minimizing resistance is particularly important in resource limited settings with limited ART options usually restricted to first-line nonnucleoside reverse transcriptase inhibitor (NNRTI)-based and second-line protease inhibitor (PI)-based regimens [6].

Consequently, access to VL and drug resistance testing in case of virological failure [VF]) is crucial to limit misdiagnosis of treatment failure which leads to undetected accumulation of resistance mutations or conversely to avoid unnecessary ART switches to more expensive ART [7]. Even if the timing of VL evaluation is still a matter of debate [8], performing VL testing at 6 months after initiating ART and every 12 months is now the preferred monitoring approach to diagnose and confirm ART failure [9]. Indeed, late diagnosis of treatment failure is associated with accumulated drug resistance mutations and high level cross resistance to subsequent regimens [10].

Retention and adherence also play a critical role in the response to ART as suboptimal viral suppression may result in higher risk of developing drug resistance [11]. In 2011, data from 149 low- and middle-income countries indicated an average retention rate of 81% at 12 months and 75% at 24 months [12]. The main critical issues for lost to follow-up patients is to differentiate self-transferred clients—when people decide to enroll in care at a new health facility without informing staff at their previous clinics—and unascertained death where vital registration data is not routinely collected in many resource limited settings [11].

Over the last decade, the Cambodia's human immunodeficiency virus (HIV) program (NCHADS, National Center for HIV/AIDS, Dermatology and STD, Sexual Transmissible Disease) has been most successful in Cambodia. The prevalence of HIV infection decreased from 2.4% in 1998 to 0.7% in 2012 [13], [14]. In December 2012, over 90% of people in need of ART are under treatment [15], leading to a total number of 50,659 treated patients, including 4,052 children 0–14 years old [16]. ART care centers are numerous, with 61 health facilities offering ART as well as drugs for opportunistic infections. VL is now recommended by the national program and is performed free of charge after 24 months, then once a year, or in case of suspicion treatment failure (based on immunologic and clinical criteria) [17].

However, data remain limited on the patterns and extent of drug resistance mutations in adults and children from this southeastern Asian country where CRF01_AE is the most common HIV strain. Most data on treatment failure for this specific HIV recombinant form were reported from Thailand, with NNRTI and NRTI resistance rates of 89–95% and 42–58% in adults and 97% and 98% in children, respectively [6], [18]–[20]. In addition, laboratories that can performed VL and DR testing are scarce outside Phnom Penh (the capital city of Cambodia). Finally, in the absence of unique identifiers for Cambodian patients, some of them, with a past experience of ART, may switch from one center to another without being detected. Data are lacking regarding the resistance profile and patterns of viral mutation of these specific patients.

The present study, conducted in Cambodia, assessed the interest of detecting VF and drug resistance at entry for new patients with ART experience. Two types of patients were considered and compared: naive patients versus pre-treated patients.

## Method

### Ethics

The survey was conducted with the authorization of the Calmette Hospital authorities. Patients enrolled in the French program “Ensemble pour une Solidarité Thérapeutique Hospitalière en Réseau” (ESTHER) cohort were recorded anonymously in the ESTHER data base. The survey was a retrospective observational anonymous survey using routine data. Patients gave oral consent to be enrolled in the ESTHER program. Patient records/information was anonymized and de-identified prior to analysis. These procedures were considered sufficient regarding ethical issues, according to Cambodian National Ethic Committee for Health Research (MoH (2001) [21].

### Study setting

This study was conducted in Calmette Hospital, a 340-bed national referral hospital located in Phnom Penh (2 million inhabitants) using ESTHER data base. ESTHER was implemented in Calmette Hospital in February 2003. It is currently taking care of 1,591 HIV patients (as of Jan 2013).

The standard first-line regimen in Cambodia at the onset of the study consisted in stavudine (d4T), lamivudine (3TC), and nevirapine (NVP) and as alternative in case of drug side effects or interaction, zidovudine (ZDV) for replacing d4T and efavirenz (EFV) for replacing NVP. TDF/3TC or abacavir (ABC)/didanosine (DDI) and lopinavir/r (LPVr) are the second line treatment regimens which are prescribed after a phase of adherence boosting for patients failing first-line treatment [17].

In terms of laboratory monitoring, HIV patients are monitored every 3 months at the outpatients department. CD4 counts are performed every 6 months. Also, since 2010, we decided to perform an early VL measurement between 6 and 12 months for ART naive patients and at inclusion for all patients who reported a previous ART experience (ART pre-treated patients).

### Study populations

From January 2010 to May 2012, all patients newly attending the HIV clinics were eligible to the study, regardless their CD4 count and clinical stage. These new patients comprised ART naive patients and those who reported previous ART experience (ART pre-treated patients). Naive patients were included if one HIV-1 RNA VL was performed during the first year of ART regimen. Patients pre-treated with ART were included if one HIV-1 RNA VL was realized during their first ESTHER consultation. Naive or ART pre-treated status was recorded according to the patients' declaration at inclusion. For ART pre-treated patients, the ART duration (in months) was retrieved from database.

### Laboratory tests

Blood samples were analyzed for CD4 counts, plasma HIV-1 RNA VL, and HIV-1 drug resistance testing at Pasteur Institute in Cambodia, as previously described [22].

Briefly, CD4 counts were obtained by flow cytometry (FACScount, Beckton Dickinson and Cyflow, Partec, Germany). Plasma HIV-1 RNA VL was performed on −80°C frozen plasma, using the Agence Nationale de Recherche sur le Sida (ANRS) second generation (G2) real-time RT-PCR test. This assay allows the quantification of HIV-1 B and non-B sub types, including the A/E CRF_01 circulating in South East Asia [23]. The threshold of the assay was 250 copies/mL, by using 200 µl of plasma.

HIV-1 genotyping was conducted for patients with a VL>1,000 copies/mL. The genotypic resistance study was done for the reverse transcriptase (RT) and protease (PR) genes and analyzed according to the 2013 version 23 ANRS algorithm [24].

### Definitions

VF was defined as plasma HIV-1 RNA level >1000 copies/mL [9].

Virological failure was compared with previous WHO immunological failure definition: CD4 cell count at 6 months below the pre-treatment level, or a 50% decline from the on-treatment peak CD4 cell count, or CD4 counts below 100 cells/µL after 12 months [25].

Multidrug resistance was defined as the presence of K65R, Q151M, or at least three TAMs [6], [26].

### Statistical analysis

Data were entered in Epi Data software. All records were cross-checked with the original data sheets. Analysis was carried out with STATA, version 9 (Stata Corporation, College Station, TX, USA). Chi squared or Fisher's exact tests were used to compare categorical variables as appropriate and Student's t-test was used to compare continuous data. Kruskal-Wallis and Wilcoxon rank sum tests were used for non-normally distributed variables. A p-value<0.05 was considered statistically significant.

## Results

### Characteristics of the populations

**Figure 1:**
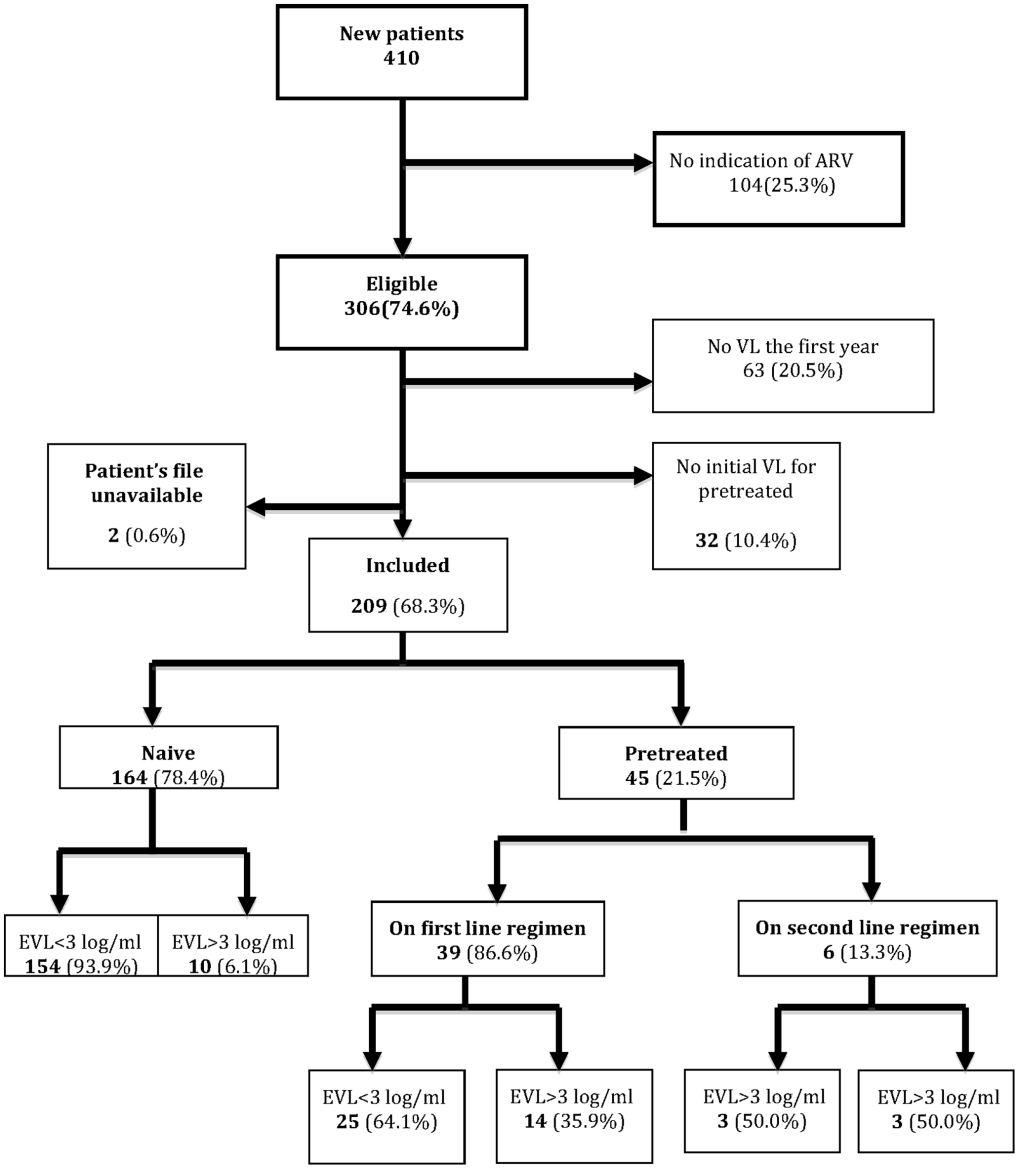
Flow chart of patients enrolled in early viral load study in Calmette hospital.

During the study period, 306 new patients were enrolled in the ESTHER program (Figure 1). Of these, 209 (68.3%) fulfilled the inclusion criteria as follows: 164 (78.4%) were ART naive patients and 45 (21.5%) had a past history of ART (pre-treated patients). Their main characteristics are described in Table 1. Naive and pre-treated patients had similar sex ratio and age. Naive patients had a lower baseline median initial CD4 cell count than pre-treated individuals (74 cells/mm3, IQR: 30–194, versus 279 cells/mm3, IQR103–455) (p<0.001), respectively.

**Table 1:** Characteristic of new patients enrolled at Calmette Hospital.

	Naive	ART Pre-treated	*P*	Total
	n = 164 (78.4%)	n = 45 (21.5%)		n = 209 (100%)
Male	85 (51.8)	23 (51.1)	*0.9*	108 (51.6)
Median age, years (IQR)	36 (30–45)	40 (32–45)	*0.1*	37 (31–45)
Phnom Penh residence	146 (89.0)	36 (80.0)	*0.1*	182 (87.0)
Median CD4 level (cells/mm^3^ (IQR))*	74 (30–194)	279 (103–455)	*<0.001*	95 (34–245)
OMS clinical stage				
<3	121 (73.7)	33 (73.3)	*0.9*	154 (73.6)
≥3	43 (26.2)	12 (26.6)		55 (26.3)
First line therapy** (n = 197)	164 (100)	39 (86.6)		203 (97.1)
-D4T containing regimen	155 (94.5)	16 (35.5)	*<0.001*	171 (81.8)
-AZT containing regimen	7 (4.2)	20 (44.4)	*<0.001*	27 (12.9)
-TDF containing regimen	1 (0.6)	1 (2.2)	*0.3*	2 (0.9)
-NVP containing regimen	96 (58.5)	27 (60.0)	*0.2*	123 (58.8)
-EFV containing regimen	67 (40.8)	11 (24.4)	*0.1*	78 (37.3)
Second line therapy*** (n = 6)		6 (13.3)		
-3TC TDF LPV/r		3 (6.6)		3 (1.4)
-AZT 3TC LPV/r		1 (2.2)		1 (0.4)
-AZT DDI LPV/r		1 (2.2)		1 (0.4)
-3TC DDI LPV/r		1 (2.2)		1 (0.4)
Median duration of ART (months)		63 (2–144)		

Numbers and (percentages). Median and (interquartile range: IQR).

D4T: stavudine, tenofovir (TDF), azidothymidine (AZT), lamivudine (3TC), nevirapine (NVP), Efavirenz (EFV), abacavir (ABC), lopinavir/r (LPVr) (/r: ritonavir as a booster), atanazavir/r (ATVr), didanosine (DDI) or darunavir (DRVr).

*at inclusion.

**Containing two nucleoside reverse-transcriptase inhibitors (NRTIs) plus either the nonnucleoside reverse-transcriptase inhibitor (NNRTI) efavirenz or Nevirapine: AZT or D4T, 3TC/FTC, NVP or EFV [26].

***Protease inhibitor based boosted regimens (LPV/RTV or ATV/RTV) [26].

As summarized in Table 1, 155 (out of a total of 164, 94.5%) naive patients received a first-line regimen including D4T. By the end of the study in December 2012, 84 (51.2%) were still under D4T. Of the 45 ART pre-treated patients, 39 (86.6%) were treated with first-line regimens whereas 6 (13.3%) with second-line regimens.

### Virological failure rates

The global rate of VF was 12.9% (95%CI: 8.6–18.2%.0%; n = 27/209). VF was higher among pre-treated patient (N = 10/164) than naive patients (N = 17/45) (6.0%, 95%CI: 2.9–10.9 versus 37.7%, 95%CI: 23.7–53.4, respectively, p<0.001) (Table 2). Similarly, among patients on first line regimen, VF was more frequent among pre-treated patients than naive patients (14, 35.8%, 95% CI: 0.2–0.5, versus 10, 6.0%, 95%CI: 2.9–10.9) (p<0001). Three subjects (out of 6) pre-treated with second-line regimens showed VF.

**Table 2:** Resistance Associated Mutations (RAMs) among naive and ART pre-treated patients with virological failure (VL>3 log/ml).

	Naive	ART Pre-exposed	Total	Pre-exposed
	First line	First line	First line	Second line
	N = 164	N = 39	N = 203	N = 6
Virological failure	10 (6.0)	14 (35.8)	24 (11.8)	3 (50)
Median Viral load, log10 copies/ml	4.3 [IQR: 3.7–4.4]	4.8 [IQR: 4.1–5.5]	-	
Genotyping test	7/10 (70.0)	12/14 (85.7)	19 (9.3)	3 (100)
At least one RAMs	7 (100)	10 (83.3)	17 (89.4)	2 (66.6)
≥3 NRTI mutations	2 (28.5)	8 (53.3)	10 (45.5)	1 (33.3)
≥3 NNRTI mutations	5 (71.4)	5 (33.3)	10 (45.5)	2 (66.6)
NRTI resistance				
3TC resistance	7 (100)	9 (75)	16 (84.2)	2 (66.6)
D4T resistance	4 (57.1)	8 (66.6)	12 (63.2)	1 (33.3)
AZT resistance	2 (20.0)	7 (58.3)	9 (47.4)	1 (33.3)
ABC resistance	1 (12.2)	3 (25.5)	4 (21.1)	0 (0)
TDF resistance	1 (14..2)	1 (8.3)	2 (10.5)	0 (0)
DDI resistance	1 (14..2)	5 (33.3)	6 (31.6)	0 (0)
NNRTI resistance				
NVP resistance	7 (100)	8 (66.6)	15 (78.9)	2 (66.6)
EFV resistance	7 (100)	8 (66.6)	15 (78.9)	2 (66.6)
ETV resistance	0 (0)	3 (25.0)	3 (15.8)	1 (33.3)
RPV resistance	0(0)	2 (16.6)	3 (15.8)	0 (0)
PI resistance				
LPVr resistance	not done	0/3 (0)		0/3 (0)
ATVr resistance	not done	0/3 (0)		0/3 (0)

Numbers and (percentages). Median and (interquartile range: IQR).

Stavudine (D4T), tenofovir (TDF), azidothymidine (AZT), lamivudine (3TC), nevirapine (NVP), efavirenz (EFV), abacavir (ABC), lopinavir/r (LPVr) (/r: ritonavir as a booster), atanazavir/r (ATVr), didanosine (DDI) or darunavir (DRVr).

A total of 10 naive patients (6.3%) presented VF while only 1 (1.0%) would have been detected using the previous immunologic WHO criteria [25].

In terms of quantitative results, median HIV-1 RNA VL for patients with VF was significantly lower (4.3 log_10_ copies/mL, IQR: 3.7–4.4) in naive patients, in comparison with pre-treated patients (4.8 log_10_ copies/mL, IQR: 4.1–5.5) (p = 0.06).

### Drug resistance

**Figure 2:**
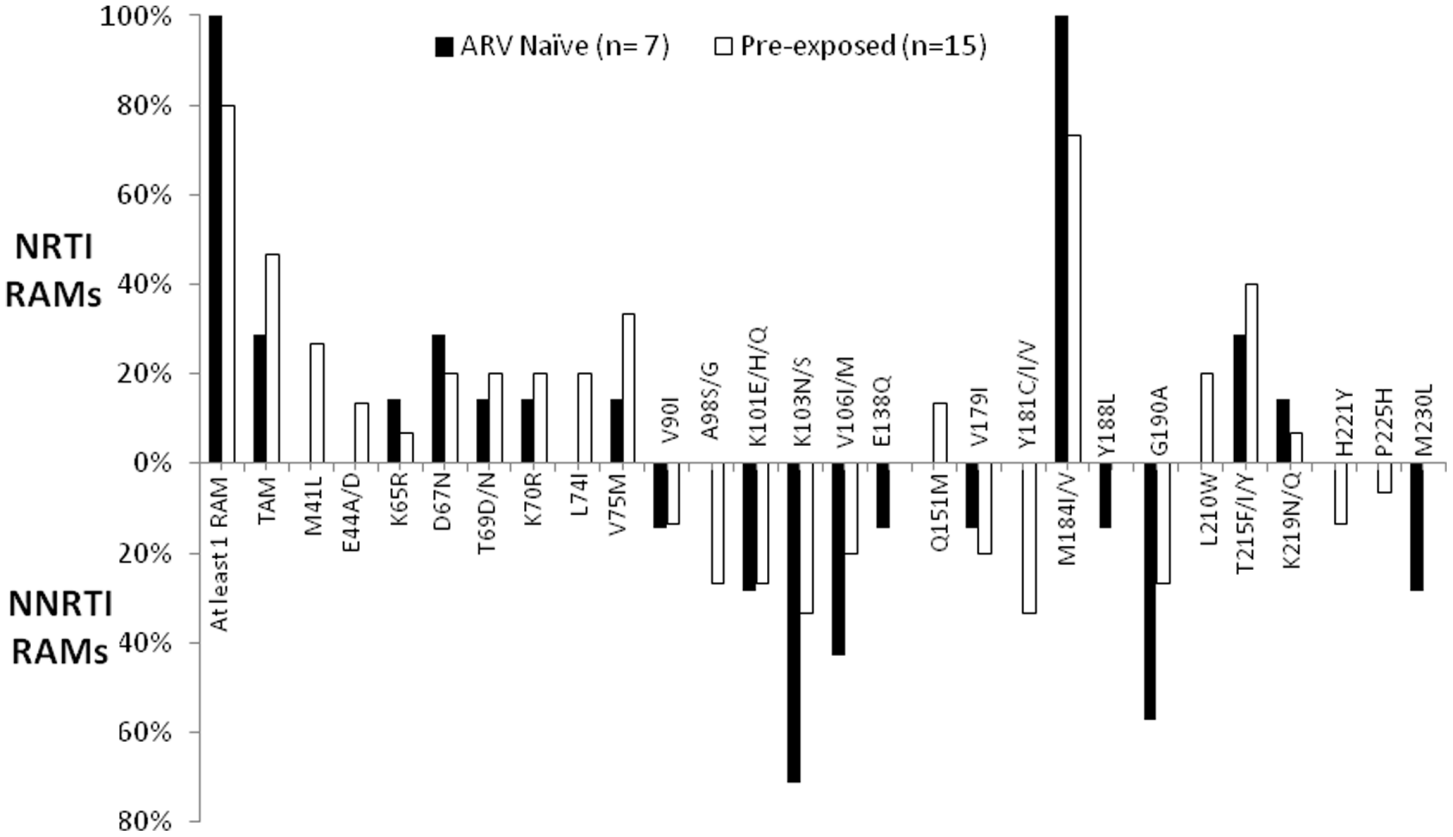
RTI-RAMs in ART naive and ART pre-treated patients with virological failure.

Of 27 patients with VF, 22 (81.4%) could be amplified and sequenced for HIV-1 PR and RT coding regions. Among these, by using the ANRS algorithm, 19 (19/22, 86.3%) patients harbored ≥1 resistance-associated mutations (RAMs). The three remaining patients harboring wild-type strains were all belonging to the pre-treated patients' category (Figure 2).

Overall, 18 (81.8%) showed dual-class resistance to NRTIs and NNRTIs (7/7, 100% vs. 11/15 (73.3%), p = 0.1, in naive and pre-treated patients, respectively). One (4.5%) harbored resistance to NNRTIs only.

The most common NRTI mutations were the M184V mutation (19/22, 86.3%). Thymidine analog mutations (TAMs) were found in (9/19, 40.9%) of patients with a higher rate in pre-treated patients, but without reaching significance. The TAM2 pathway (D67N, K70R, T215F, and K219Q/E) was present in 5/22, 4/22, 8/22, 2/22 patients. The TAM1 pathway was present mostly in pre-treated patients (M41L 4/15, 27.7%; L210W: 3/15, 20%, T215Y/I/Y: 6/15, 40%). V75T/M and T69D/N mutations, associated with decreased susceptibilities to d4T and ddI, were detected in 4/22 (18.1%) and 6/22 (27.2%) patients. L74V/I/IL was seen in 20% of pre-treated patients, not in naive patients. Together with K65R, this mutation is associated with resistance to ddl, ABC, and TDF. Multi-Nucleos(t)ide Resistance (MNR) were only observed in pre-treated patients, including 2 (13.3%) harboring Q151M, K65R, E33A/D, E44A/D mutations.

Regarding the NNRTI mutations, V90L, K101E, K103N/S, V106I/M, V1791L were observed in both patients groups. However, K103N tend to be more frequent in naive patients than in pre-treated patients (n = 5, 71.4% versus n = 5, 33.3%), (p = 0.09). The Y181C/I (N = 5, 33.3%), A98S/G (n = 4, 27%), H221Y (n = 2, 13%) and P225H (N = 1, 7.5%) mutations were only seen in pre-treated patients. In five pre-treated patients, viruses were found resistant to etravirine (ETR), a second-generation NNRTI drug, due to the simultaneous presence of the Y181C and G190A mutations.

In terms of resistance to drugs, among the 7 naive patients, viruses were found resistant to 3TC/FTC (100%), D4T (57.1%), AZT (20.0%), ABC, DDI and TDF (12.2%), NVP/EFV (100%) and RPV (28.5%) (Table 3). Finally, all tested-naive were resistant to 3TC/FTC/NVP/EFV. All but one remained sensitive to didanosine (DDI), ABC, TDF, and all were sensitive to ETR.

**Table 3:** Frequency of mutation by molecule in naive and ART pre-treated patients with virological failure.

Patients Category	Patients	HIV-1 RNA VL (Log/mL)	NRTI						NNRTIs			
			ZDV	D4T	3TC/FTC	ABC	DDI	TDF	NVP	EFV	ETR	RVP
ARV-naïve patients	10-1806	3,7	**R**	**R**	**R**	*PR*	*PR*	*PR*	**R**	**R**	**SS**	*N/A*
	10-1701	4,5	**R**	**R**	**R**	*PR*	*PR*	*PR*	**R**	**R**	*PR*	*N/A*
	10-1866	3,4	*PR*	**R**	**R**	*PR*	*PR*	*PR*	**R**	**R**	*PR*	**R**
	10.1828	4,3	*PR*	**R**	**R**	*PR*	*PR*	*PR*	**R**	**R**	**SS**	**R**
	11.2005	3,7	*PR*	*PR*	R	*PR*	*PR*	*PR*	**R**	**R**	*PR*	*PR*
	11.1919	4,8	*PR*	*PR*	R	*PR*	*PR*	*PR*	**R**	**R**	*PR*	*PR*
	10.1872	4,4	*PR*	*PR*	R	*PR*	*PR*	*PR*	**R**	**R**	*PR*	*N/A*
ARV pre-exposed patients	12.2095	5,8	**R**	**R**	**R**	**R**	**R**	**R**	**R**	**R**	*PR*	**R**
	10-1779	4,6	**R**	**R**	**R**	**R**	**R**	**R**	**R**	**R**	*PR*	*N/A*
	10-1885	5,5	PR	**R**	R	PR	*PR*	*PR*	**R**	**R**	**R**	*N/A*
	10-1896	4,4	**R**	**R**	**R**	**SS**	R	**SS**	**R**	**R**	*PR*	*N/A*
	10-1830	5,4	**R**	**R**	**R**	PR	*PR*	*PR*	**R**	**R**	**R**	*N/A*
	10-1796	4,8	**R**	**R**	**R**	**R**	**R**	**SS**	**R**	**R**	**SS**	*N/A*
	10-1754	5,6	**R**	**R**	**R**	**SS**	PR	**SS**	**R**	**R**	*PR*	*N/A*
	1759	4,1	**R**	**R**	**R**	**SS**	**R**	**SS**	*PR*	*PR*	**R**	*N/A*
	11.1990	5,5	*PR*	*PR*	**R**	**R**	**R**	*PR*	**R**	**R**	PR	PR
	10-1836	5	*PR*	*PR*	*PR*	*PR*	*PR*	*PR*	**SS**	*PR*	*PR*	*N/A*
	1991	4,2	*PR*	*PR*	*PR*	*PR*	*PR*	*PR*	*PR*	*PR*	*PR*	*N/A*
	10-1900	3,5	*PR*	*PR*	*PR*	*PR*	*PR*	*PR*	*PR*	*PR*	*PR*	*N/A*
	11-1963	6,4	*PR*	*PR*	*PR*	*PR*	*PR*	*PR*	*PR*	*PR*	*PR*	*N/A*
	10-1876	3,4	*PR*	*PR*	**R**	*PR*	*PR*	*PR*	**R**	**R**	**R**	*N/A*
	2002	4,1	**R**	**R**	**R**	*PR*	**SS**	**SS**	**R**	**R**	**R**	PR

**R** = Resistant, *PR* = Possible resistant, **SS** = sensitive.

For pre-treated patients on first line regimen, HIV strains were resistant to 3TC (75%), D4T (66.6%), AZT (58.3%), ABC (25.5%), TDF (8.3%), NVP/EFV (66%), rilpivirine (RPV) and etravirine (ETV) (16.6%). Among pre-treated patients on second-line regimens, none harbored viruses showing resistance to lopinavir/r (LPV/r), atanazavir/r (ATV/r) or dolutegravir (DRV/r), two were resistant to TPVr and one to indinavir (IDV).

Finally, four ART pre-treated patients were still sensitive to first-line ART.

## Discussion

Our study conducted in Cambodia showed a high level of VF among new pre-treated patients at their initial consultation (35.8% and 50.0% for those under 1^st^ line and 2^nd^ line regimens, respectively). By contrast, the 12 months VF rate of naive patients was quite low (6.0%). In addition, among naive patients with virological ART failure, resistance mutations to 3TC and NNRTI appeared quickly and were generalized to all patients. For pre-treated patients, TAMs were frequently observed.

The fact that naive patients showed a very low level of VF supports the efficacy of the 1^st^ line treatment, and emphasizes the need on regular monitoring of patients' adherence. This result is consistent with previous reports of studies conducted one to four years after the onset of first-line ARV regimen in Cambodia [27]–[31]. The VF rate observed after 4 year of ART was similar (4%) in another Phnom Penh hospital [30]. Interestingly, the VF rate reported herein is lower than that reported in sub-Saharan Africa patients where up to 24% of VF is obtained within 12 months of initiation of first-line ART [32].

The presence of early mutations in the naive patient group conferring resistance to NNRTI and in a lesser extent to NRTI is of concern. All naive patients harbored viruses that were resistant to NVP/EFV with a majority of them showing the K103N/S mutation. The rates of 181C/I, K103N/S G190A and M184L/V mutations were also higher than in another recent study in Cambodia among patients having a median duration of 12 months up to 4 years on antiretroviral therapy [6], [7], [30]. The level of transmitted drug resistance (TDR) in naive patients needs to be assessed and could explain the high level of resistance mutations for this population potentially more recently infected than pre-treated patients. Finally, the number of TAM was limited and TDF option was preserved as second-line regimen backbone for all but one naive patients.

By contrast, failure rate among ART pre-treated patients was high in comparison with a recent multisite study involving several African and Asian countries where levels of VF ranged between 2.9% and 20.6% after one year [33]. In our experience, the proportion of ART pre-treated patients represented roughly 22% of new HIV patients admitted in Calmette Hospital. The reason for seeking treatment at Calmette Hospital was not documented in the patient's files. However, it may be related to a well known ART failure or ART breakthrough in the previous center explaining the high VF rate. Another possible reason is that patients previously treated in the private sector returned to the Calmette center in order to benefit with free drugs provided by the national program. This raises the concern of patients' nomadism and the absolute need of a network between ART care units. The need for patient-unique identifiers is also required because it will help in avoiding misreporting and in increasing the proper allocation of treatment for patients. This issue is currently under consideration within the national Cambodian program.

In pre-treated patients, the high frequency of TAM mutations may be explained by the extensive use of thymidine analogues in resource limited settings. The accumulation of TAMs might reduce the effectiveness of second-line TDF-containing combinations which are recommended in Cambodia [17]. However, among ART second-line regimen VF patients, all were sensitive to LPV/r, ATV/r and TDF. This is consistent with evidence that suggests that adherence support is critical for these patients [34]. A recent study conducted among ART second-line VF in Cambodia report that two-thirds of patients present no resistance mutation for protease and need only adherence support [35]. A national evaluation is currently being conducted for patients treated with second-line regimen in Cambodia.

Finally, it must be pointed out that three pre-treated patients showed VF but carried HIV strains that had no mutation at all. In our setting, we think that early transient viremia may be associated with a reversion to wild type viruses. As a consequence, among pre-treated patients, it is likely that some of them experienced unstructured treatment interruptions, thus resistance would not be identified as frequently.

### Study limitations

Our survey has some limitations. First, it is a retrospective study suffering from the usual limitations of retrospective approaches. Also, the interpretation of data must be cautious due to small sample size. Second, blood samples prior to ART initiation were not available. Thus, the potential occurrence of transmitted drug resistance (TDR) could not be investigated [1], [36]. The rate of transmitted drug resistance is around 7.6% in Asia [17], [37]. Third, it was not possible to document the impact of adherence and the duration of viral failure among pre-treated patients. A previous study in the same facility estimated the adherence at 88% and 81% among naive and pre-treated patients, respectively [38]. Fourth, around twenty percent of the new patients had no VL during the first year and were excluded from the study. This may have led to underestimation of treatment failure.

## Conclusion

The emergence of drug resistance mutations seems to occur relatively quickly, notably to NNRTI, in naive patients. Also, specific attention should be paid to ART pre-treated patients in whom resistance mutations to NRTI were relatively common. VL must be provided early in the follow up of ART treated patients. HIV genotypic assays before ART initiation and for ART pre-treated patients infection should be considered as well. This will avoid increasing treatment failures, morbidity and mortality and unnecessary switching to expensive second or even third-line therapies.
